# Erianin Targets PINK1/Parkin‐Mediated Mitophagy and Apoptosis to Ameliorate Atopic Dermatitis

**DOI:** 10.1002/fsn3.72158

**Published:** 2026-07-24

**Authors:** Kexin Xu, Lianhua Zhu, Shan Jin, Wenyu Jin, Zhehu Jin, Guanghai Yan, Liangchang Li

**Affiliations:** ^1^ Jilin Key Laboratory for Immune and Targeting Research on Common Allergic Diseases Yanbian University Yanji The People's Republic of China; ^2^ Department of Anatomy, Histology and Embryology Yanbian University Medical College Yanji The People's Republic of China; ^3^ Department of Dermatology Affiliated Hospital of Yanbian University Yanji The People's Republic of China; ^4^ Key Laboratory of Natural Medicines of the Changbai Mountain, Ministry of Education Yanbian University Yanji The People's Republic of China

**Keywords:** atopic dermatitis, erianin, mitochondrial apoptosis, mitophagy, PINK1

## Abstract

Atopic dermatitis (AD) is a chronic inflammatory skin lesions. Mitochondrial dysregulation is associated with various pathologic conditions, including inflammation in the skin. Prior research has indicated that Erianin, a naturally occurring compound derived from Dendrobium plants, has antioxidant and anti‐inflammatory effects. Generally, Erianin has been demonstrated to be effective in cutaneous melanoma and ulcerative colitis. However, there is limited knowledge regarding its biological components and the mechanisms by which it prevents and treats AD. To investigate the protective effects of Erianin against AD and to elucidate the potential mechanism of inflammation in AD, with a particular focus on the role of mitochondrial impairment pathways. Combined with vivo and vitro models of inflammation was employed to assess skin conditions, oxidative stress, and mitochondrial dysfunction. The mitochondrial homeostasis was analyzed, and the effects of Erianin treatment on these processes were evaluated using histological analysis, biochemical assays, molecular techniques, and targeting inflammatory cytokines, oxidative stress, mitophagy, and apoptosis. Erianin effectively alleviated AD, and computational simulations suggested a potential interaction with PINK1. Treatment with Erianin significantly reversed these dysfunctional states by promoting mitophagy activity, thereby alleviating mitochondrial dysfunction and reducing ROS levels, which ultimately suppressed apoptosis. These findings demonstrate that Erianin exerts therapeutic potential in AD by targeting mitophagy, with PINK1/Parkin‐mediated mitophagy playing a crucial role in the pathogenesis of AD.

## Graphical abstract

Erianin activates PINK1/Parkin‐mediated mitophagy, reduces ROS accumulation, inhibits mitochondrial apoptosis, and alleviates skin inflammation in atopic dermatitis.

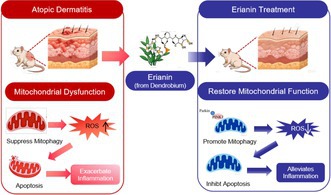

## 1 Introduction

Atopic dermatitis (AD) is a common chronic immunoinflammatory skin disorder with pruritus and squamous lesions but relapsing processes. The hyperfunction of Th2 cells is a crucial reason for AD (Yang et al. 2020). AD has been growing under social pressures, with other atopic diseases such as rhinitis and asthma (Marques et al. 2024). This has occurred all period from children to adults. The majority of therapies focus on relieving symptoms; with the introduction of drugs like Janus kinase inhibitors and corticosteroids, the issue of relapse following the cessation of these medications continues to be a major concern (Eichner and Wohlrab 2022). Therefore, it is crucial to identify new safe and effective agents that can improve AD exacerbations with minimal side effects.

Mitochondria also play a vital role in enabling the skin to effectively maintain epidermal homeostasis (Quan et al. 2025). Reactive oxygen species (ROS) are generated from mitochondria. Accumulating evidence suggests a correlation between ROS and mitochondrial aberrations evident and has been identified as a significant feature of inflammatory and allergic diseases such as AD (Rizwan et al. 2020). AD exacerbates oxidative stress by disrupting cellular redox homeostasis, decreasing antioxidant capacity, and causing mitochondrial dysfunction, which ultimately triggers apoptosis (Raimondo et al. 2023). Advanced research has shown that under pathological conditions, the PINK1/Parkin‐mediated mitophagy pathway is impaired, leading to the accumulation of damaged mitochondria, increased oxidative stress, and dysregulation of inflammation and apoptosis (Liu, Wang, et al. 2022; Wang et al. 2021). Its activation promotes mitophagy and regulates oxidative stress, mitochondrial dysfunction, and apoptosis (Liu, Guo, et al. 2022). Of note, the mechanisms of PINK1 activating Parkin are still unclear; a recent study reported that PINK1 phosphorylates Parkin, which still needs to be elucidated. Besides, the role of PINK1/Parkin in AD regulating the progression of disease is still unclear.

Erianin is a bibenzyl compound extracted from Dendrobium chrysotoxum, and has been reported to exert immune regulation, anti‐proliferation, antioxidant, and anti‐inflammatory properties (Zhang et al. 2021). Mo et al. proposed that Erianin inhibited proliferation and regulated apoptosis of HaCaT cells by ROS (Mo et al. 2019). However, the pharmacologic action and molecular mechanism of Erianin on AD are barely understood. Considering that ROS, mitochondrial aberrations, abnormal apoptosis, and proliferation are involved in the development of AD, and that Erianin induces the inhibition of proliferation and regulation of ROS (Szymański et al. 2021). However, neither the mechanism nor the effect of Erianin on AD has been reported.

In the present study, we examined the role of Erianin in the inflammatory processes of AD via the Pink1/Parkin pathway. We hypothesize substantial effects of Erianin on mitochondrial function through the PINK1/Parkin pathway and cell survival as well as on the prevention of oxidative stress in skin cells, as well as represent a promising therapeutic approach to counteracting AD.

## 2 Materials and Methods

### 2.1 Animals

Forty adult female BALB/c mice (aged 6–8 weeks) were supplied by the Laboratory Animal Center of Yanbian University. All experimental procedures were approved by the Institutional Animal Care and Use Committee (IACUC) of Yanbian University (Approval No. YD20240530003). Mice were anesthetized with isoflurane during skin sensitization/challenge. Euthanasia was performed by cervical dislocation under deep anesthesia on day 31.

### 2.2 Animal Treatment and Grouping

**FIGURE 1:**
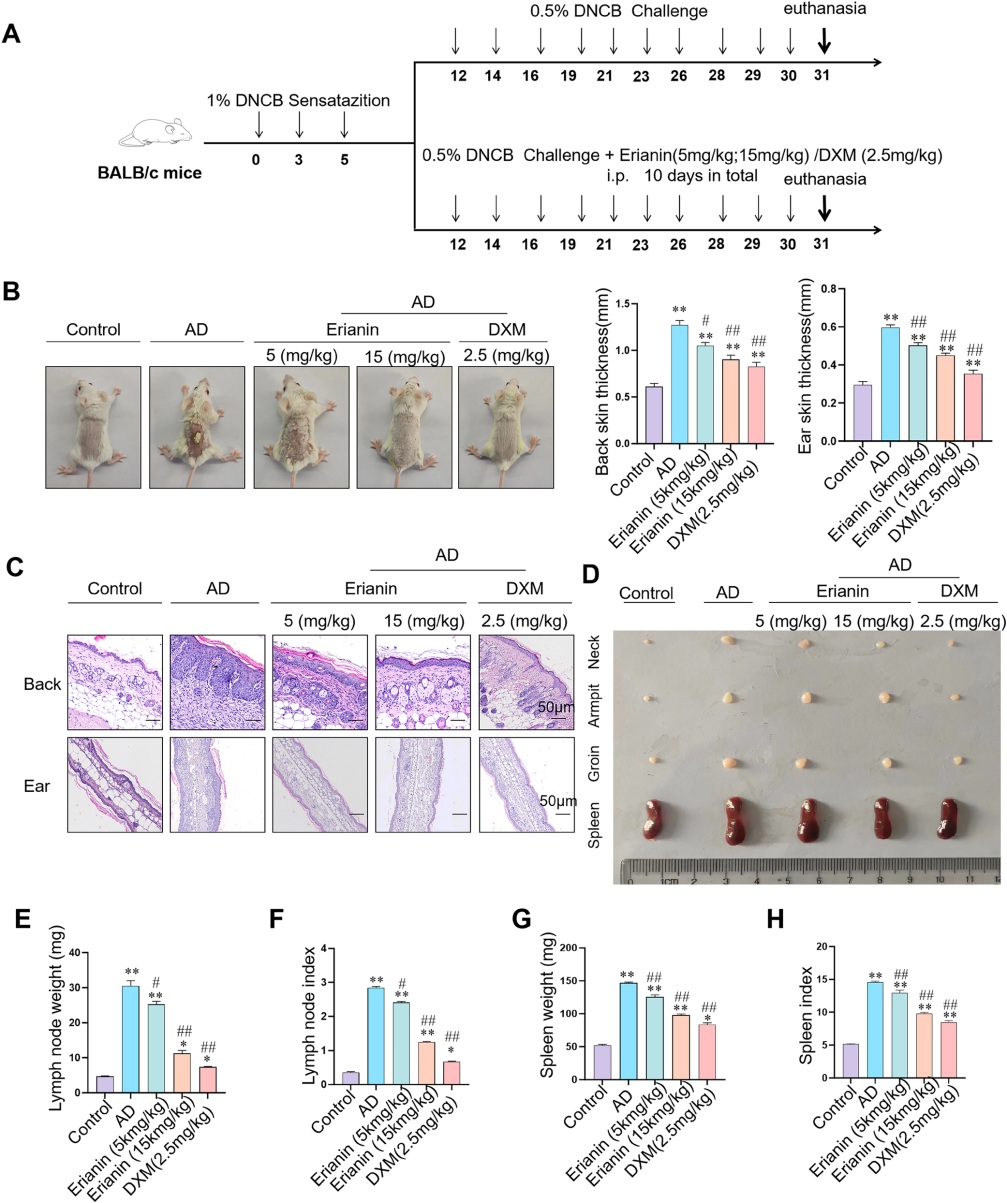
Erianin improves the pathological conditions of skin, lymph nodes, and spleen in AD mice. (A) Schematic diagram of AD model establishment. (B) Macroscopic skin changes and measurement of dorsal/auricular skin thickness on day 31. (C) H&E staining of dorsal and ear skin. Scale bar = 50 μm. (D) Gross morphology of lymph nodes and spleen. (E–H) Quantification of lymphoid organ changes: (E) Lymph node weight, (F) Lymph node index, (G) Spleen weight, (H) Spleen index. The data are expressed as the mean ± SD. (*n* = 8) One‐way ANOVA with Student's *t*‐test was used to perform the comparison of means. *p < 0.05, **p < 0.01, compared to the Control group; ^#^ *p* < 0.05, ^##^ *p* < 0.01, compared with the AD group.

Forty mice were randomly allocated into five groups (the Control, DNCB + vehicle (AD), DNCB + Erianin (Erianin, 5 mg/kg), DNCB + Erianin (Erianin, 15 mg/kg) and DNCB + dexamethasone (DXM, 2.5 mg/kg)) (Zeng et al. 2022). To induce AD‐like inflammation, 1‐chloro‐2,4‐dinitrobenzene (DNCB, 237329‐10G; Alorich Merck, NJ, USA) was diluted in a 3:1 mixture of acetone and olive oil. 1% DNCB was used in the sensitization phase, followed by repeated challenge with 0.5% DNCB. For the sensitization process, bilateral ear (20 μL) and back skin (200 μL) were topically treated on days 0, 3 and 5. After 1 week of sensitization, mice were challenged with 0.5% DNCB at the same sites every other day from day 12 to day 30 while on Erianin and DXM. To test the effections of Erianin (B20844; Shanghai Yuanye Biotechnology Co Ltd., Shanghai, China) response, Mice received intraperitoneal (i.p.) injection of 5 mg/kg and 15 mg/kg before 0.5% DNCB treated from day 12 to day 30, respectively. The DEX group received intraperitoneal (i.p.) injection of 2.5 mg/kg. The Control and AD model groups received i.p. injections of an equal volume of saline at the same time points to serve as vehicle controls. On day 31, all groups of the mice were euthanized and necropsied. The experimental design is summarized in Figure 1A.

### 2.3 Cell Culture and Drug

HaCaT cells (CL0114‐21; Fenghui Biotechnology, Changsha, China) were procured and cultured in DMEM medium which contained 10% fetal bovine serum (FBS, 040011ACS‐50 mL; Biological Industries, Kibbutz Beit Haemek, Israel), 1% penicillin–streptomycin 100 U/mL penicillin. Cells were incubated in a humidified atmosphere with a temperature of 37°C and 5% CO_2_.

For experimental conditions, cells were incubated in following mediums: To establish the TNF‐α‐induced inflammatory model, the culture medium was replaced, and HaCaT cells were exposed to 10 ng/mL TNF‐α for a duration of 24 h. As Erianin treatment groups, cells were incubated in two different culture mediums: a 1 nM Erianin medium and a 4 nM Erianin medium for 2 h, and then stimulated with 10 ng/mL TNF‐α (300‐01A‐10UG; Peprotech, Princeton, USA) for 24 h, in the presence of Erianin. Erianin (B20844; Shanghai Yuanye Biotechnology Co. Ltd., Shanghai, China) was dissolved in dimethyl sulfoxide (DMSO) and then diluted with culture medium to the indicated concentrations. The final concentration of DMSO in all treatment groups, including the control group, was maintained below 0.1% (v/v). Control cells were treated with an equivalent volume of DMSO (vehicle control) under the same conditions to exclude any potential solvent effects on cell viability and experimental outcomes.

To investigate the role of PINK1 in mitochondrial damage, siRNA targeting PINK1 (sc‐44,598; Santa Cruz Biotechnology, Dallas, TX, USA), 5′‐GAGUAGCCGCAAAUGUGCUUCAUCU‐3′, and one negative control, non‐specific siRNA, were used for specific silencing (13,778,150, Invitrogen, USA). Cells transfected with these si‐RNAs using Santa Cruz. Liposome RNAiMAX reagent (13,778,150, Invitrogen, USA). HaCaT cells (8 × 10^3^cells/well) were cultured in six‐well plates followed by transient transfection for 48 h with PINK1 siRNA or NC siRNA after pretreatment of cells with Erianin for 2 h.

### 2.4 Measurement of the Lymph Nodes, Spleen, Ear Thickness, and Epidermal Thickness

To evaluate the tissues and organs of each mouse was obtained on day 31. The ear and back skin thickness of the mice were assessed using a thickness gauge and documented. The weights of the lymph nodes and spleen were assessed, and the organ indices were computed for both based on Equation: Organ index: (organ weight/mouse body weight) × 100%.

### 2.5 Hematoxylin and Eosin (H&E)

At the end of the study, Mice ear and dorsal skin tissues of randomly selected area in three sections for mice were excised for histological examination, tissues were harvested and fixed in 4% phosphate‐buffered formalin for 24 h. The formalin‐fixed tissues were embedded in paraffin, sectioned, and stained with H and E (G1122‐100 mL; Solarbio, Beijing, China). Images were analyzed using a slide scanning system (SQS‐40R; Shengqiang Technology, Shenzhen, China).

### 2.6 Flow Cytometry

Mouse spleens and lymph nodes were minced, and live‐dead dye (65–0865‐14; Invitrogen, Carlsbad, California, USA) was introduced, followed with incubated at 4°C for 30 min. Live cells were isolated, stained with the surface antibody anti‐CD4 (mouse) monoclonal antibody labeled with FITC (D341‐4; MBL, Woburn, MA, USA) at 4°C, and incubated for 30 min. Subsequently, a membrane disruption solution was applied, and the cells were stained with intracellular antibodies including APC‐Rat Anti‐Mouse IL‐4 (554,436, BD Biosciences San Diego, California, USA) and PE‐Rat Anti‐Mouse IL‐13 (568,551, BD Biosciences San Diego, California, USA) gamma monoclonal antibody. Detect cell apoptosis using an apoptosis assay kit (BD Biosciences, CA, USA) and analyze it using a flow cytometer (Cytoflex, Beckman Coulter Inc., CA, USA).

### 2.7 Enzyme‐Linked Immunosorbent Assay (ELISA) for Detection of Cytokines

ELISA Kits (MULTISCIENCES; Guangzhou, China; PEPROTECH, Princeton, USA) were used according to the manufacturer's instructions to detect the levels of thymic stromal lymphopoietin (TSLP) (EK265; MULTISCIENCES; Guangzhou, China), interleukin‐33 (IL‐33) (EK233; MULTISCIENCES; Guangzhou, China), interleukin‐25 (IL‐25) (BMS6046TEN; PEPROTECH, Princeton, USA), and interleukin‐17 (IL‐17) (900‐K392; PEPROTECH, Princeton, USA) in skin tissues. Harvested the supernatant and centrifugated at 4°C, 3500 rpm for 20 min, then measured the levels of cytokines as protocol described.

### 2.8 Microarray Profiling and Data Preprocessing

To isolate total RNA, which included 3 ad lesional samples and 3 control samples, were employed for external validation. Raw data (CEL files) were background‐corrected and normalized using the RMA algorithm via Agilent Feature Extraction Software (v11.0.1).

### 2.9 Differential Expression Analysis

Differentially expressed genes (DEGs) were identified using the limma package (v3.56.2) in R with thresholds of |log2 fold change (FC)| ≥ 1 and adjusted *p*‐value < 0.05. Volcano plots were generated using the Weishengxin Online Platform (https://www.bioinformatics.com.cn) by uploading the DEG list (log2FC and *p*‐values).

### 2.10 Enrichment Analysis of Gene Ontology (GO) Terms and KEGG Pathway

Gene Ontology (GO) terms analysis was performed to elucidate genetic regulatory networks of interest by forming hierarchical categories through the biological process (BP), cellular component (CC), and molecular function (MF) aspects of the DEGs (http://www.geneontology.org). Pathway analysis was performed to obtain the significant pathways of the DEGs through the KEGG (http://www.genome.jp/kegg/). A *p*‐value cut‐off of.

### 2.11 Quantitative Real‐Time RT‐PCR

Total RNA was extracted from mouse dorsal skin tissues using TRIzol reagent (Invitrogen, USA) following the manufacturer's instructions. Complementary DNA (cDNA) synthesis was carried out via reverse transcription using the PrimeScript RT Reagent Kit (Takara, Japan). Real‐time PCR was conducted with the LightCycler 480 System (Roche, Switzerland). Gene expression levels were normalized to mouse Gapdh, and relative gene expression was calculated using the 2^−ΔΔCt^ method. All primer sequences utilized in the analysis are detailed in Table 1.

**TABLE 1:** Sequences of primers.

Genes		Sequences (5′‐3′)
*Pink1*	Forward	CAGCGCTACCGTGAGGAAAT
	Reverse	CCGATCACTGCCCATCTTGA
*Parkin*	Forward	TGGGCCGTCATGAACTTCAT
	Reverse	TGCCGAATCATACTTGGCCT
*Gapdh*	Forward	AGGTCGGTGTGAACGGATTTG
	Reverse	TGTAGACCATGTAGTTGAGGTCA

### 2.12 Immunohistochemistry (IHC)

The Tissue sections were then incubated overnight at 4°C with a rabbit monoclonal anti‐Cleaved‐Caspase‐3 antibody (#9661S; GST, Guangzhou, China). After washing, a horseradish peroxidase (HRP)‐conjugated anti‐rabbit polymer enhancer (PV‐9000; OriGene Technologies, Rockville, MD, USA) was applied dropwise and incubated for 60 min at room temperature. Color development was performed using diaminobenzidine substrate for 2–5 min, followed by hematoxylin counterstaining. Digital images were acquired using a high‐resolution slide scanner (SQS‐40R; Shengqiang Technology, Shenzhen, China).

### 2.13 MTT Assay

The effects of erianin on the cytotoxicity of HaCaT cells were assessed by MTT (0.5 mg/mL, C0009S, Beyotime, China). Cells were located at a concentration of 8 × 10^3^cells/well in a 96‐well plate incubated overnight, and then treated with various concentrations of erianin (0, 1, 2, 4, 8, and 10 nM) for 24 h. Erianin was dissolved in DMSO and then diluted with culture medium to the indicated concentrations. To control for solvent effects, the final concentration of DMSO was kept consistent (below 0.1%) across all treatment groups, including the 0 nM control group. After treatment, 0.02 mL MTT solution was added per well for another 4 h, and then the DMSO (150 μL/well) (D8371, Solarbio, China) was added. The solution was shaken to dissolve the purple formazan crystals and allowed to react in an incubator for 10 min. The absorbance was read at 490 nm on a microplate reader (BioTek, Winooski, VT, USA).

### 2.14 Mitochondrial Membrane Potentia (MMP) and ROS Measurement

To investigate MMP, a mitochondrial membrane potential kit (JC‐1, C2006; Beyotime, Shanghai, China) was used. HaCaT cells were seeded into culture plates and treated with two concentrations of Erianin for 24 h. Cells were treated with the JC‐1 solution at 25°C for 20 min. Fluorescence analysis was carried out through a Cytation5 instrument (BioTek, Winooski, VT, USA).

For the detection of ROS, skin, ear tissues, and cells were measured by DHE Assay Kits (# S0063, Beyotime, Shanghai, China) and ROS Assay Kits (#S0033S, Beyotime, Shanghai, China). Immunofluorescence detection of skin and ear tissues was performed through a Cytation5 instrument. HaCaTs were washed three times, suspended in serum‐free medium, and incubated at 37°C. The cells were washed and then imaged under flow cytometry (Cytoflex, Beckman Coulter Inc., CA, USA).

### 2.15 TUNEL

Tissue and cell apoptosis were assessed using the TUNEL Apoptosis Kit (C1091; Beyotime, Shanghai, China) and the One‐Step TUNEL Apoptosis Detection Kit (C1089; Beyotime, Shanghai, China). For tissue sections, dehydration was performed using a graded ethanol series, followed by treatment with DNase‐free proteinase K (20 μg/mL) for 15 min at 37°C. Then, 50 μL of TUNEL reaction mixture was applied to the sections and incubated in the dark at 37°C for 60 min. Color development was achieved with diaminobenzidine (DAB) chromogen for 5 min, followed by counterstaining with hematoxylin and mounting with neutral resin. Finally, images were captured and analyzed using a digital slide scanner (SQS‐40R; Shengqiang Technology, Shenzhen, China).

Cell Apoptosis Detection. For cell TUNEL assays, cells were fixed with 4% paraformaldehyde for 20 min, permeabilized with 0.5% Triton X‐100 for 10 min on ice, and incubated with 100 μL TUNEL reaction solution at 37°C for 30 min in the dark. Fluorescent signals were detected using a Cytation 5 imaging multimode reader (BioTek, Winooski, VT, USA).

### 2.16 Immunofluorescence

The treated Hacat was fixed with methanol at 4°C for 20 min, blocked with 5% BSA at room temperature for 1 h, and then permeated with 0.2% Triton for 30 min. After incubation at room temperature for 30 min with MitoTracker Red CMXRos (M7512, Invitrogen, Carlsbad, CA, USA) and overnight staining with PINK1 (DF7742, affinity, Jiangsu, China), Parkin (A0968, abclonal, Wuhan, China) primary antibody, the corresponding Alexa Fluor 488 goat anti‐rabbit secondary antibody (R37118, Life Technologies, Waltham, Massachusetts, USA) was incubated at 37°C for 2 h. Fluorescence images were captured under Cytation 5.

### 2.17 Western Blot

Electrophoresis was conducted using 10% SDS‐PAGE, followed with transfer to a PVDF membrane and sealing with milk powder. The membranes were then probed with primary antibodies, such as Nrf2 (SAB4501984, Sigma‐Aldrich, St. Louis, Missouri, USA), HO‐1 (ab189491, Abcam, Cambridge, England, UK), PINK1 (DF7742, affinity, Jiangsu, China), Parkin (A0968, abclonal, Wuhan, China), Bcl‐2 (AF6139, affinity, Jiangsu, China), Cleaved‐Caspase‐3 (9661S, CST, Danvers, Massachusetts, USA), and β‐actin (3700 s, CST, Danvers, Massachusetts, USA) overnight at 4°C. After that, incubation with secondary antibodies was conducted for 1 h and 30 min at 25°C. ECL was utilized for color development, and membranes were visualized using Quantity One software (BioRad, Hercules, CA, USA). Protein expression levels were quantified with Image J.

### 2.18 Molecular Docking

The protein structures of PINK1 were obtained from the Protein Data Bank (PDB) database. Molecular docking studies were conducted using AutoDockTools‐1.5.7, 3D visualization was performed utilizing PyMOL, and 2D image analysis was carried out with LigPlus.

### 2.19 Statistical Analysis

Statistical analysis was performed utilizing Prism 10.0 software, and the results are expressed as mean ± standard deviation. Normality was assessed using the Shapiro–Wilk test, and homogeneity of variances was assessed using Levene's test prior to one‐way analysis of variance (ANOVA). Group comparisons were evaluated using *t*‐tests (for two groups) or one‐way ANOVA followed by Tukey's post hoc test (for multiple groups). Statistical significance was considered for *p* < 0.05.

## 3 Results

### 3.1 Erianin Improves the Pathological Conditions of Skin, Lymph Nodes and Spleen in AD Mice

To investigate the therapeutic effect of Erianin on AD, a DNCB‐induced AD mouse model was established (Figure 1A). Compared with the Control group, the AD group exhibited significantly increased dorsal and auricular skin thickness, along with characteristic AD lesions including hemorrhage, edema, and epidermal desquamation. Treatment with Erianin or DXM significantly reduced both dorsal/auricular skin thickness and the severity of AD‐like lesions compared to the AD group (*p* < 0.01) (Figure 1B). H&E‐stained dorsal skin and ear sections revealed that Erianin treatment significantly decreased epidermal thickness in AD mice (Figure 1C). Furthermore, evaluation of lymphoid organs demonstrated that both the absolute weights and the organ indices (organ weight/body weight) of the lymph nodes and spleen were significantly lower in the Erianin and DXM groups than in the AD group (*p* < 0.01) (Figure 1D–H). Collectively, these results demonstrate that Erianin ameliorates DNCB‐induced skin thickening and reduces lymph node and spleen enlargement in AD mice.

### 3.2 Erianin Suppresses Pro‐Inflammatory Cytokine Production in AD Mice

**FIGURE 2:**
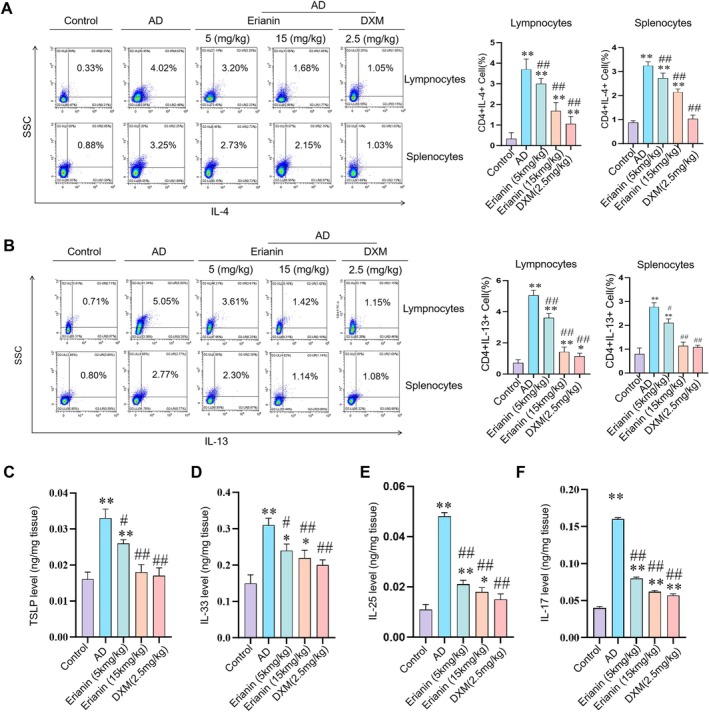
Erianin suppresses pro‐inflammatory cytokine production in AD mice. (A) Flow cytometry analysis and quantification of IL‐4^+^CD4^+^ T cells in lymph nodes and splenocytes. (B) Flow cytometry analysis and quantification of IL‐13^+^CD4^+^ T cells in lymph nodes and splenocytes. (C–F) ELISA quantification of cytokines in skin homogenates: (C) TSLP, (D) IL‐33, (E) IL‐25, (F) IL‐17. The data are expressed as the mean ± SD (*n* = 8). One‐way ANOVA with Student's *t*‐test was used to perform the comparison of means. *p < 0.05, **p < 0.01, compared to the Control group; ^#^ *p* < 0.05, ^##^ *p* < 0.01, compared with the AD group.

Flow cytometry analysis revealed significantly lower levels of IL‐4 and IL‐13 in lymph nodes and splenocytes in both the Erianin groups and DXM group compared to the AD group (*p* < 0.01) (Figure 2A,B). Furthermore, ELISA assays demonstrated significantly decreased levels of TSLP, IL‐33, IL‐25, and IL‐17 in skin homogenates in the Erianin and DXM groups compared to the AD group (*p* < 0.01) (Figure 2C–F). Collectively, these results indicate that Erianin effectively suppresses the production of key AD‐associated pro‐inflammatory cytokines in mice.

### 3.3 Transcriptomic Profiling Reveals Dysregulated Autophagy and Apoptosis in AD Mice

**FIGURE 3:**
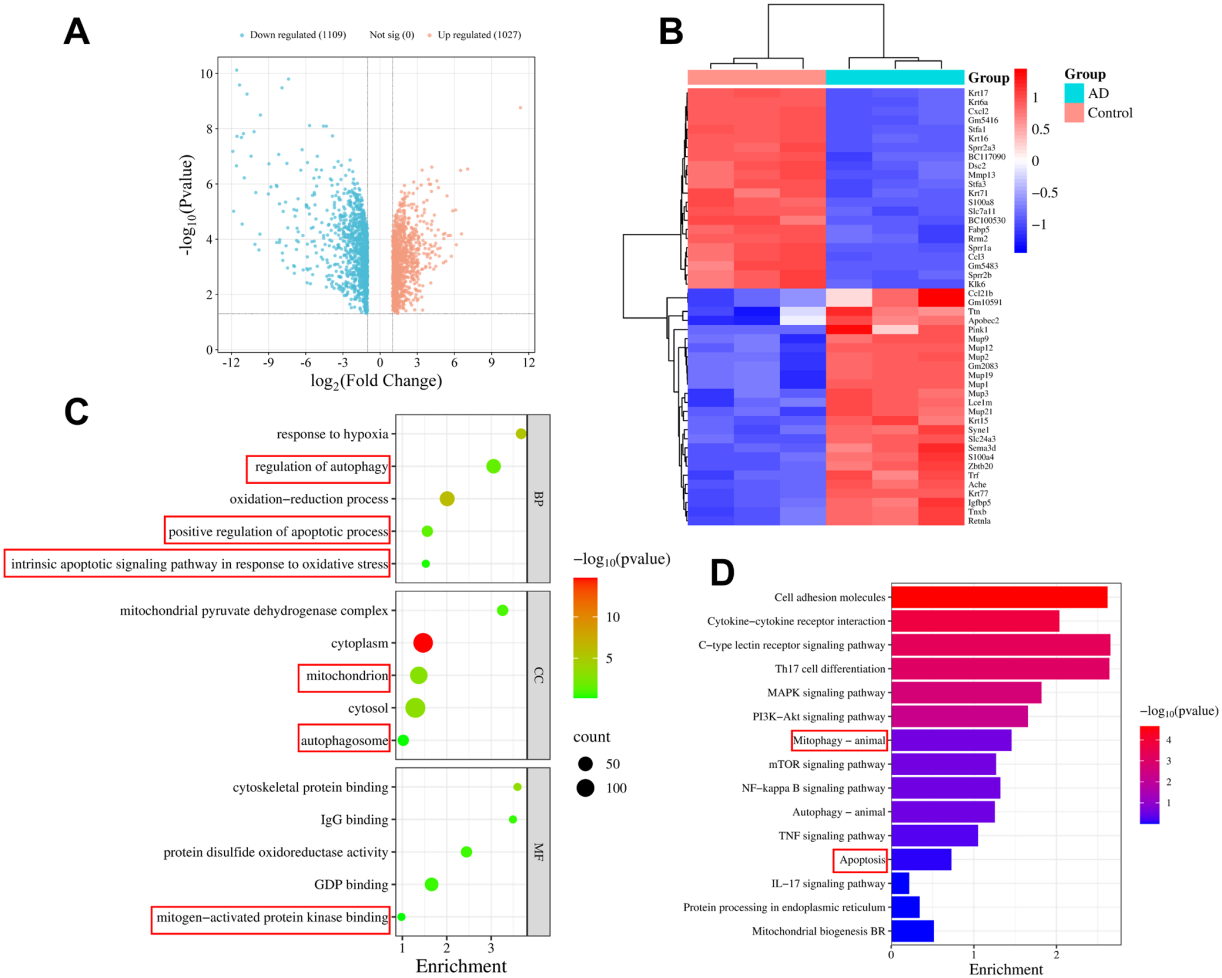
Transcriptomic profiling reveals dysregulated autophagy and apoptosis in AD mice. (A) Volcano plot of DEGs (Control vs. AD). (B) Hierarchical clustering of DEGs. (C) GO enrichment analysis. (D) KEGG enrichment analysis (*n* = 3).

Microarray and cluster analysis revealed 1109 downregulated and 1027 upregulated genes in the AD group compared to the control group (Figure 3A,B). GO enrichment analysis of the (DEGs) showed that the most significantly enriched (BP) were related to oxidative stress, apoptotic, and autophagic processes. CC terms were predominantly associated with mitochondria and autophagosomes.MF terms were chiefly involved in mitogen‐activated protein kinase‐binding (Figure 3C). KEGG pathway analysis further revealed that the signaling pathways most significantly enriched among the DEGs were primarily associated with autophagy and apoptosis (Figure 3D). Collectively, these findings suggest that dysregulation of autophagy and apoptosis may contribute to AD pathogenesis.

### 3.4 Erianin Reduces ROS Accumulation and Enhances PINK1/Parkin Signaling to Attenuate Apoptosis in AD Mice

**FIGURE 4:**
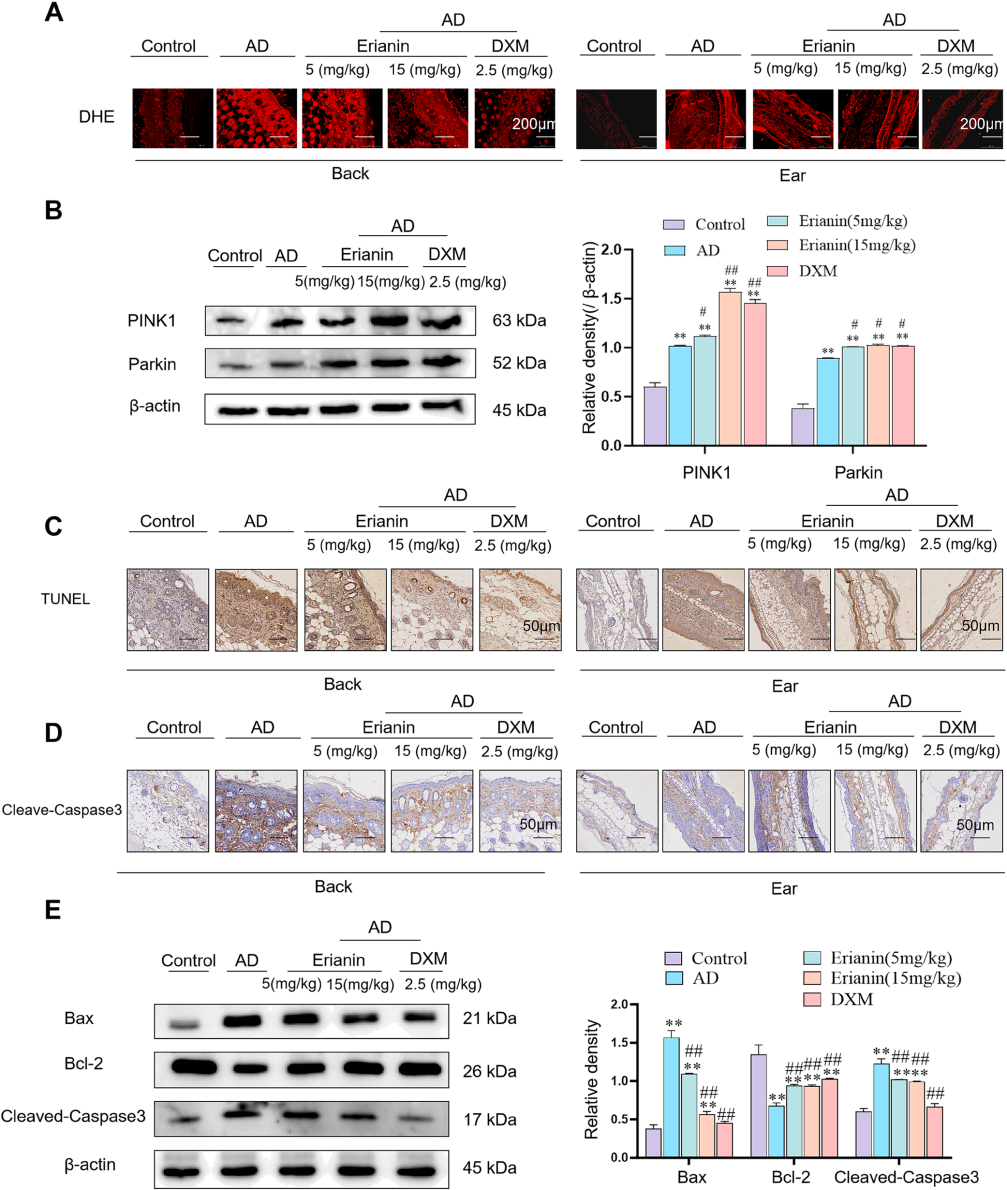
Erianin reduces ROS accumulation and enhances PINK1/Parkin signaling to attenuate apoptosis in AD mice. (A) DHE assays back skin and ears for ROS generation. Scale bar = 200 μm. (B) Left: Western blot of PINK1/Parkin. Right: Quantification (normalized to β‐Actin). (C) TUNEL of back and ears tissue. Scale bar = 50 μm. (D) Immunohistochemistry of Cleaved‐Caspase‐3. Scale bars = 50 μm. (E) Left: Western blot of Bax/Bcl‐2/Cleaved‐Caspase‐3. Right: Quantification (normalized to β‐Actin). The data are expressed as the mean ± SD (*n* = 3). One‐way ANOVA with Student's *t*‐test was used to perform the comparison of means. *p < 0.05, **p < 0.01, compared to the Control group; ^#^ *p* < 0.05, ^##^ *p* < 0.01, compared with the AD group.

We assessed ROS levels in mouse ears and dorsal skin. DHE staining showed significantly reduced ROS accumulation in the Erianin and DXM groups compared to the AD group (*p* < 0.01) (Figure 4A). To investigate whether Erianin regulates PINK1/Parkin, we performed qRT‐PCR to examine *PINK1* and *Parkin* mRNA expression in mouse skin tissues. The results showed that compared with the AD group, the Erianin and DXM groups exhibited significantly increased *PINK1* and *Parkin* mRNA expression (*p* < 0.05) (Figure S1). Western blot results were consistent with the changes in mRNA expression (Figure 4B). TUNEL assays demonstrated significantly decreased apoptosis in the ears and dorsal skin of Erianin and DXM groups compared to the AD group (*p* < 0.01) (Figure 4C). Immunohistochemistry confirmed reduced Cleaved‐Caspase‐3 expression in Erianin and DXM groups compared to the AD group (*p* < 0.01) (Figure 4D). Western blot results indicated a decrease in Cleaved‐Caspase‐3 and Bax protein expression levels, along with an increase in Bcl‐2 expression level following Erianin and DXM groups (*p* < 0.01) (Figure 4E). Collectively, Erianin may attenuate apoptosis associated with enhanced PINK1/Parkin signaling in AD mice.

### 3.5 Erianin Attenuates Oxidative Stress and Mitochondrial Dysfunction in TNF‐α‐Induced HaCaT Cells

**FIGURE 5:**
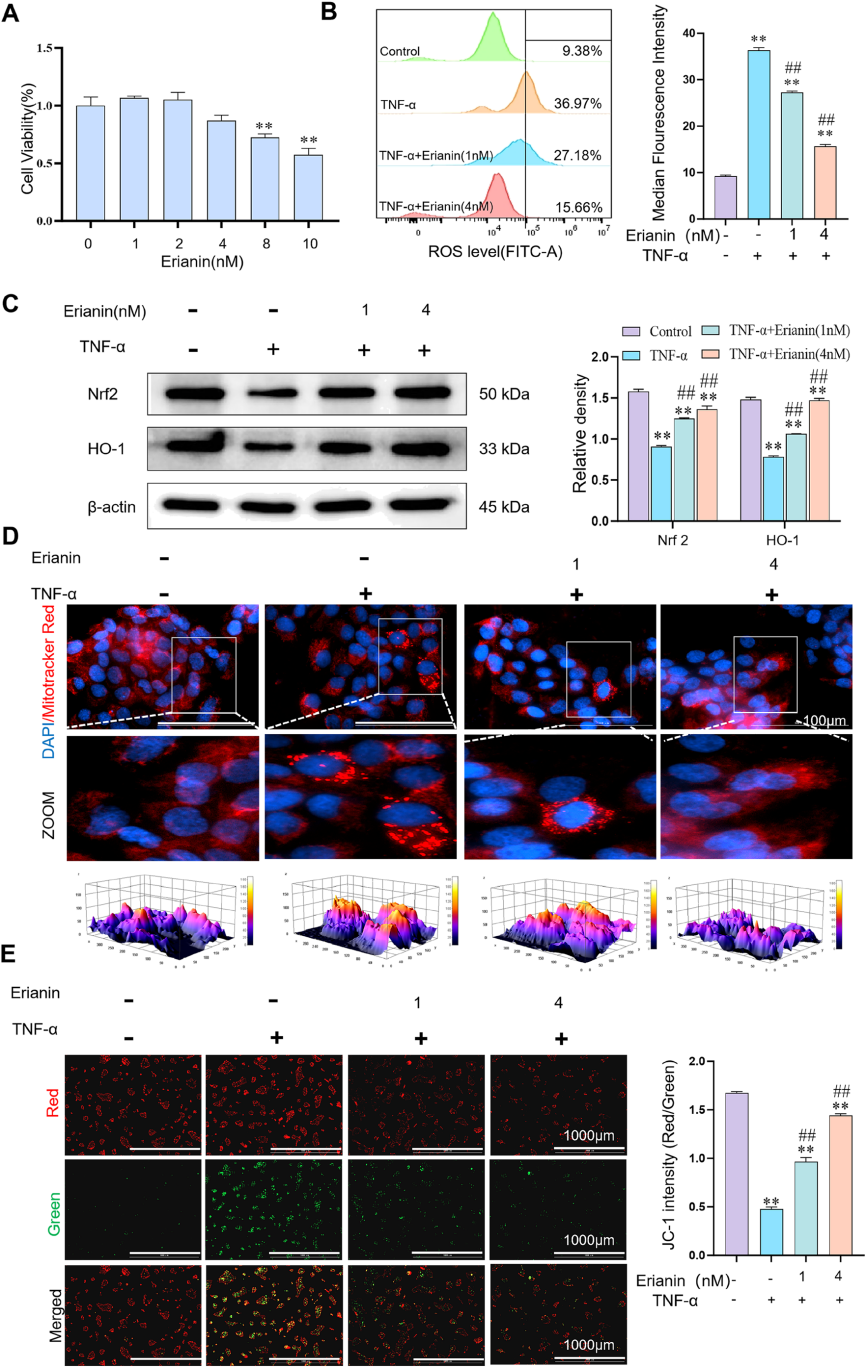
Erianin attenuates TNF‐α‐induced oxidative stress and mitochondrial dysfunction in HaCaT cells. (A) Cell viability (MTT assay). (B) Flow cytometry detection of ROS. (C) Left: Western blot of Nrf2/HO‐1. Right: Quantification (normalized to beta;‐Actin). (D) Mitochondrial morphology (MitoTracker Red). Scale bars = 100 μm. (E) Mitochondrial membrane potential (JC‐1). Scale bar = 1000 μm. The data are expressed as the mean ± SD (*n* = 3). One‐way ANOVA with Student's *t*‐test was used to perform the comparison of means. *p < 0.05 **p < 0.01, compared to the Control group; ^#^ *p* < 0.05 ^##^ *p* < 0.01, compared with the TNF‐α group.

MTT assays showed no cytotoxicity of Erianin (1–4 nM) compared to control (*p* < 0.01) (Figure 5A). Flow cytometry revealed Erianin significantly reduced intracellular ROS levels in TNF‐α‐stimulated cells (*p* < 0.01) (Figure 5B). Western blot demonstrated upregulated HO‐1 and Nrf2 expression after Erianin treatment (*p* < 0.05) (Figure 5C). Immunofluorescence with MitoTracker Red showed Erianin preserved mitochondrial integrity (Figure 5D). JC‐1 assays indicated Erianin restored MMP, evidenced by increased red/green fluorescence ratio versus TNF‐α‐only group (*p* < 0.05) (Figure 5E). Collectively, Erianin may protect against mitochondrial dysfunction by enhancing HO‐1/Nrf2 and suppressing ROS.

### 3.6 Erianin Activates the PINK1/Parkin Pathway Through Binding to PINK1

**FIGURE 6:**
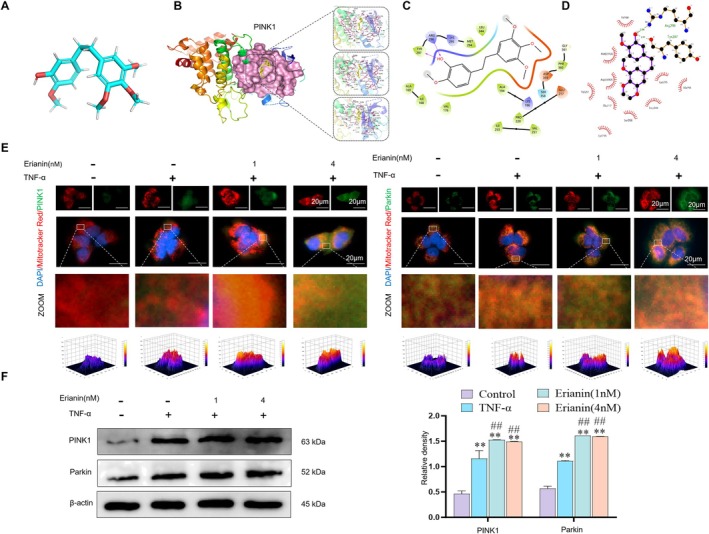
Erianin activates the PINK1/Parkin pathway through binding to PINK1. (A) The chemical structures of Erianin of 3D. (B) The docking result between Erianin and PINK1 in 3D. (C, D) The docking result between Erianin and PINK1 in 2D. (E) Immunofluorescence staining of PINK1 (green, left) and Parkin (green, right) in HaCaT cells. Scale bars = 20 μm. (F) Left: Western blot of PINK1/Parkin. Right: Quantification (normalized to β‐Actin). The data are expressed as the mean ± SD. (*n* = 3) One‐way ANOVA with Student's *t*‐test was used to perform the comparison of means. *p < 0.05 **p < 0.01, compared to the Control group; ^#^ *p* < 0.05 ^##^ *p* < 0.01, compared with the TNF‐α group.

Molecular docking revealed Erianin binds PINK1 with a docking score of −5.84 kcal/mol, forming hydrogen bonds with ARG296 and TYR297 residues (Figure 6A–D). Given that decreased MMP activates PINK1/Parkin‐mediated mitophagy, we examined this pathway. Immunofluorescence showed TNF‐α stimulation increased PINK1/Parkin expression in HaCaT cells, which was further enhanced by Erianin treatment (Figure 6E). Western blot confirmed Erianin significantly upregulated both PINK1 and Parkin protein levels compared to TNF‐α‐only stimulation (*p* < 0.05) (Figure 6F). These findings demonstrate Erianin activates the PINK1/Parkin pathway through direct binding to PINK1.

### 3.7 Erianin Inhibits Apoptosis of HaCaT Cells Induced by TNF‐α

**FIGURE 7:**
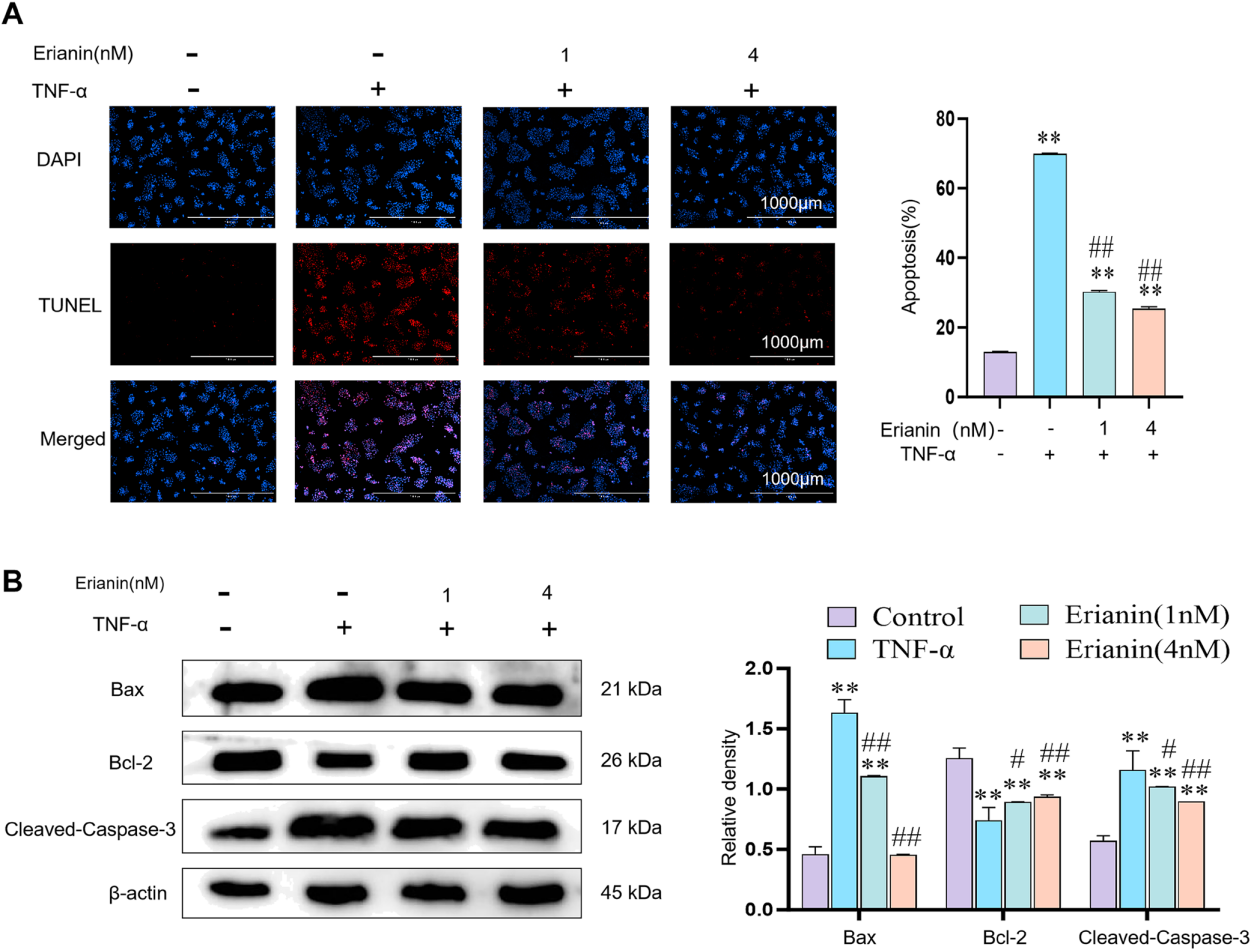
Erianin inhibits apoptosis of HaCaT cells induced by TNF‐α. (A) TUNEL assay was performed to evaluate cell apoptosis. Scale bar = 1000 μm. (B) Left: Western blot of Bax/Bcl‐2/Cleaved‐Caspase‐3. Right: Quantification (normalized to β‐Actin). The data are expressed as the mean ± SD (*n* = 3). One‐way ANOVA with Student's *t*‐test was used to perform the comparison of means. *p < 0.05, **p < 0.01, compared to the control group; ^#^ *p* < 0.05, ^##^ *p* < 0.01, compared with the TNF‐α group.

To investigate Erianin's anti‐apoptotic effects in TNF‐α‐stimulated HaCaT cells, TUNEL assays showed significant reduction of apoptosis in Erianin groups compared to the TNF‐α group (*p* < 0.05) (Figure 7A). Western blot analysis confirmed Erianin downregulated pro‐apoptotic proteins (Bax and Cleaved‐Caspase‐3) while upregulating the anti‐apoptotic Bcl‐2 (*p* < 0.05) (Figure 7B).

### 3.8 Erianin Inhibits ROS and Apoptosis via the PINK1/Parkin Signaling Pathway

**FIGURE 8:**
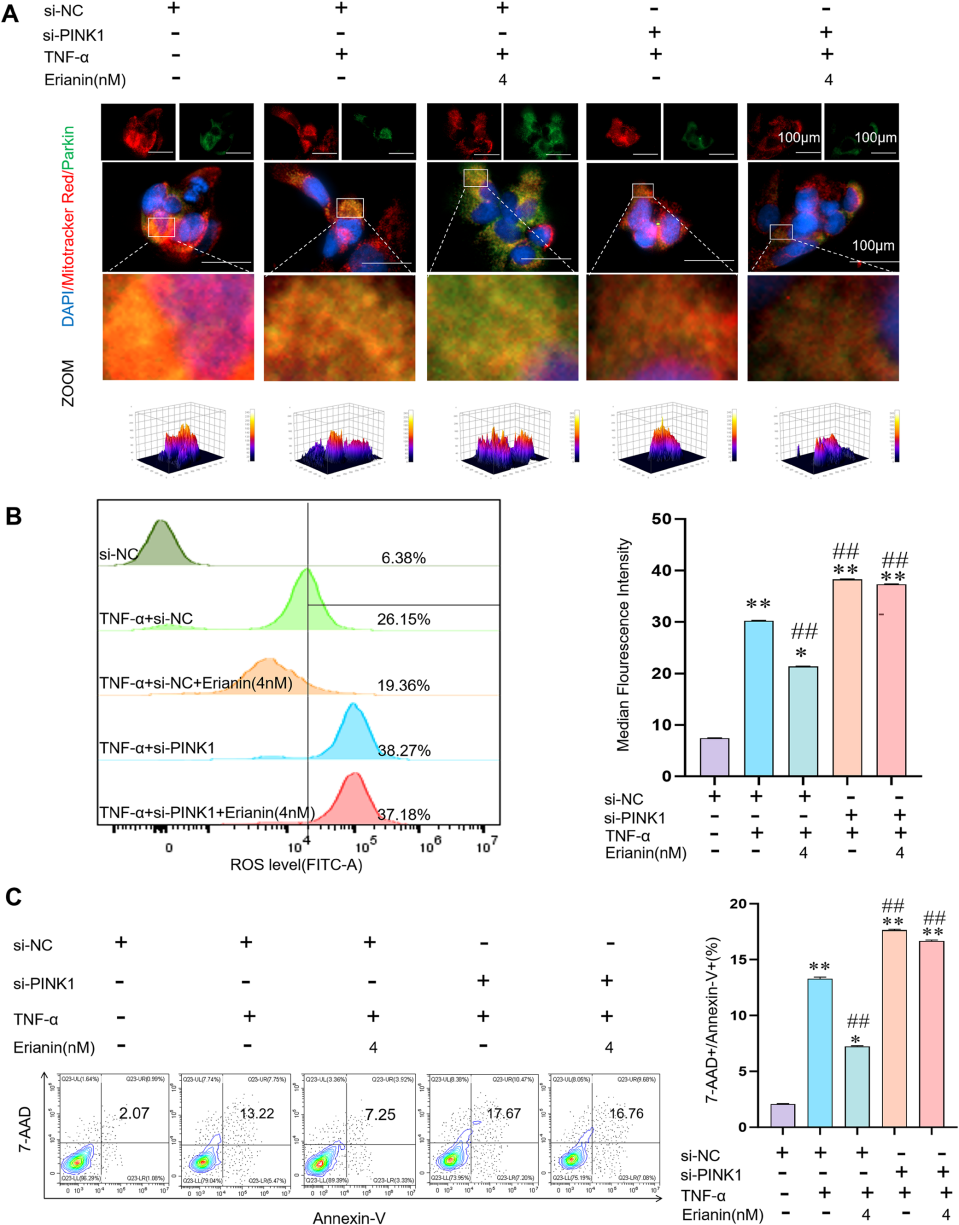
Erianin inhibits ROS and apoptosis via the PINK1‐Parkin signaling pathway. (A) Immunofluorescence of Parkin (green) after PINK1 silencing. Scale bars = 100 μm. (B) Flow cytometry detection of ROS. (C) Apoptosis rate by flow cytometry. The data are expressed as the mean ± SD (*n* = 3). One‐way ANOVA with Student's *t*‐test was used to perform the comparison of means. *p < 0.05 **p < 0.01, compared to the si‐NC group; ^#^ *p* < 0.05 ^##^ *p* < 0.01, compared with the TNF‐α+si‐NC group.

To validate PINK1's role in Erianin's mechanism, we silenced PINK1 using siRNA. Immunofluorescence analysis revealed significantly reduced Parkin expression following PINK1 knockdown (*p* < 0.01) (Figure 8A). ROS analysis demonstrated that the TNF‐α + si‐NC + Erianin (4 nM) group exhibited significantly lower ROS levels compared to the TNF‐α + si‐NC group. Conversely, the TNF‐α + si‐PINK1 group showed significantly higher ROS levels relative to the TNF‐α + si‐NC group (*p* < 0.01). Notably, no significant difference in ROS levels was observed between the TNF‐α + si‐PINK1 group and the TNF‐α + si‐PINK1 + Erianin (4 nM) group (Figure 8B). Flow cytometry analysis of apoptosis revealed parallel effects: Apoptosis was significantly reduced in the TNF‐α + si‐NC + Erianin (4 nM) group compared to the TNF‐α + si‐NC group (*p* < 0.01). In contrast, apoptosis was significantly increased in the TNF‐α + si‐PINK1 group relative to the TNF‐α + si‐NC group (*p* < 0.01). Importantly, Erianin treatment (4 nM) failed to reduce apoptosis in the TNF‐α + si‐PINK1 group, as evidenced by the lack of significant difference between the TNF‐α + si‐PINK1 group and the TNF‐α + si‐PINK1 + Erianin (4 nM) group (Figure 8C). Collectively, these data demonstrate that PINK1 is required for Erianin to suppress both ROS accumulation and apoptosis, indicating that the anti‐apoptotic effect of Erianin is dependent on an intact PINK1/Parkin‐mediated mitophagy pathway.

## 4 Discussion

Atopic dermatitis (AD) is a chronic inflammatory skin disorder characterized by predominant Th2‐type immune responses (Gandhi et al. 2016). Topical glucocorticoids remain a cornerstone treatment for AD; however, their long‐term use is associated with significant local adverse effects, such as skin atrophy, purpura, telangiectasia, and pigmentation (Maubec et al. 2015). Therefore, exploring alternative therapeutic agents is crucial. In this study, we investigated the natural compound Erianin as a potential alternative for AD management, using DXM as a positive control. Our findings demonstrate that Erianin significantly ameliorated DNCB‐induced AD‐like lesions in mice, as evidenced by reduced skin thickening and suppressed production of key inflammatory cytokines (IL‐4, IL‐13, IL‐25, IL‐33, IL‐17, and TSLP). Mechanistically, Erianin activated PINK1/Parkin‐mediated mitophagy, attenuated oxidative stress, and inhibited apoptosis in both mouse models and TNF‐α‐stimulated HaCaT cells.

Mitophagy is a crucial cellular process that ensures the health and function of the mitochondrial network by selectively removing damaged, aged, or dysfunctional mitochondria (Zhang et al. 2023). This process plays a vital role in maintaining cellular energy homeostasis and reducing oxidative stress (Ajoolabady et al. 2022). Mitophagy is primarily regulated by the PINK1/Parkin pathway, which is extensively studied (Thayer et al. 2026). Within this pathway, stimulation by inflammatory factors prevents PINK1 translocation to the inner mitochondrial membrane, leading to its accumulation on the outer membrane. (Jin et al. 2010). Consequently, PINK1 accumulates on the outer mitochondrial membrane, triggering the recruitment and activation of Parkin. PINK1‐phosphorylated Parkin, functioning as an E3 ubiquitin ligase, mediates the ubiquitination of mitochondrial substrates (Shiba‐Fukushima et al. 2012). This ubiquitination promotes the assembly of the phagophore membrane around the mitochondria, leading to autophagosome formation. Subsequent fusion with lysosomes results in degradation, completing mitophagy (Swerdlow and Wilkins 2020). Our findings indicate that Erianin increases the expression of PINK1 and Parkin in both skin tissues of AD mice and TNF‐α‐stimulated HaCaT cells. These results suggest that Erianin might mitigate AD pathology by activating PINK1/Parkin‐mediated mitophagy.

Having established that Erianin promotes mitophagy, we next examined its effect on oxidative stress. Oxidative stress promotes intracellular ROS accumulation and subsequent mitochondrial dysfunction, ultimately triggering mitochondrial apoptosis (Rizwan et al. 2020). Nrf2, a master regulator of the antioxidant response, controls the expression of cytoprotective enzymes like HO‐1 (Kim et al. 2017). The HO‐1 system plays a pivotal role in counteracting oxidative stress, protecting MMP by mitigating oxidative damage. Supporting its antioxidant potential, Erianin has demonstrated anti‐inflammatory and antioxidant effects in a model of glucosamine sodium sulfate‐induced ulcerative colitis, mediated through modulation of TLR4 and STAT3 pathways (Dou et al. 2020). Consequently, scavenging excessive ROS represents a viable strategy to mitigate inflammation. Consistent with this, our data show that Erianin significantly attenuated ROS accumulation in both the skin and ear tissues of AD mice and reduced ROS production in TNF‐α‐stimulated HaCaT cells. Therefore, Erianin‐induced mitophagy likely facilitates the clearance of dysfunctional mitochondria, thereby reducing ROS levels and alleviating oxidative stress in AD.

Accumulating evidence indicates that mitochondrial dysfunction and excessive ROS production are not merely downstream consequences of inflammation but active contributors to AD pathogenesis (Natarelli et al. 2024). In keratinocytes, oxidative stress impairs tight junction proteins (e.g., claudin‐1, occludin) and disrupts epidermal differentiation, leading to skin barrier dysfunction—a hallmark of AD (Koch et al. 2023). Furthermore, damaged mitochondria release damage‐associated molecular patterns (DAMPs) such as mitochondrial DNA, which can activate keratinocytes and dendritic cells to promote Th2‐type immune responses, including the production of IL‐4, IL‐13, and TSLP (Lin et al. 2022). Recent studies have directly linked mitophagy defects to AD pathogenesis, showing that disruption of PINK1/Parkin‐mediated mitophagy exacerbates skin inflammation and barrier dysfunction (Yue et al. 2025). By enhancing PINK1/Parkin‐mediated mitophagy, Erianin facilitates the clearance of dysfunctional mitochondria, thereby reducing ROS levels. This effect may explain the observed improvement in skin barrier integrity and the suppression of Th2 cytokines in our AD mouse model. Thus, the therapeutic benefit of Erianin in AD likely stems from its ability to break the vicious cycle between mitochondrial dysfunction, barrier disruption, and type 2 inflammation.

Our findings suggest that Erianin inhibits apoptosis indirectly by promoting mitophagy. Erianin treatment increased PINK1/Parkin expression, restored MMP, and reduced ROS levels, indicating that its primary role is to activate mitophagy and facilitate the clearance of damaged mitochondria. This removal of dysfunctional mitochondria eliminates the upstream signals that would otherwise activate the intrinsic apoptotic pathway, leading to reduced Caspase‐3 activation and cell death. When mitophagy was disrupted by PINK1 knockdown, the protective effect of Erianin against apoptosis was completely abolished. This loss of effect demonstrates that the anti‐apoptotic action of Erianin is not direct, but rather a consequence of improved mitochondrial quality control achieved through functional mitophagy.

To gain structural insights into Erianin's potential interaction with the PINK1/Parkin pathway, we performed molecular docking studies to investigate the binding of Erianin to PINK1. Molecular docking simulates interactions between PINK1 and Erianin based on their structural characteristics. Our docking results predict a potential binding interaction between Erianin and PINK1, which is consistent with our experimental observation of Erianin‐induced upregulation of PINK1/Parkin expression and mitophagy activation. This interaction could facilitate the clearance of damaged mitochondria, contributing to reduced ROS levels and, ultimately, the alleviation of skin inflammation in AD via the inhibition of mitochondrial apoptosis.

Although this study provides new experimental evidence for the therapeutic effects and underlying mechanisms of Erianin in AD, several limitations should be acknowledged. At the molecular level, molecular docking predicted a potential interaction between Erianin and PINK1 with a docking score of −5.84 kcal/mol. However, this finding is based solely on computational simulation and does not constitute sufficient evidence for direct binding, which requires further validation using biophysical techniques such as surface plasmon resonance or isothermal titration calorimetry. At the animal model level, the DNCB‐induced mouse model used in this study recapitulates the acute inflammatory features of AD but does not fully reflect its chronic and relapsing nature in humans. Moreover, the absence of systematic assessments of scratching behavior, dynamic changes in skin lesion severity, and disease recurrence after drug withdrawal limits a comprehensive evaluation of Erianin's therapeutic efficacy throughout the disease course. Regarding dose exploration and in vivo safety, although MTT assays established the safe concentration range of Erianin in HaCaT cells and provided a foundation for in vitro mechanistic studies, the extrapolation from these in vitro concentrations to in vivo doses lacks pharmacokinetic support. Furthermore, systematic evaluations of Erianin's bioavailability, metabolic stability, acute toxicity, and long‐term effects on skin tissue are currently lacking—data essential for assessing its clinical translation potential. Finally, the present findings are derived solely from preclinical models without validation in human tissues or clinical trials. In summary, this study provides a theoretical foundation for the potential use of Erianin in treating AD. Future research should focus on further elucidating its molecular mechanisms, improving in vivo safety assessments, and exploring its clinical applicability.

Taken together, our results indicate that Erianin alleviates the inflammatory response in AD by suppressing ROS generation, promoting PINK1/Parkin‐mediated mitophagy, and inhibiting apoptosis.

## Author Contributions

**Kexin Xu:** conceptualization, validation, formal analysis, writing – original draft, investigation. **Wenyu Jin:** conceptualization, formal analysis, validation. **Liangchang Li:** conceptualization, data curation, supervision, resources, writing – review and editing. **Zhehu Jin:** conceptualization, methodology. **Lianhua Zhu:** conceptualization, funding acquisition, project administration. **Shan Jin:** conceptualization, visualization, software. **Guanghai Yan:** conceptualization, validation, resources, supervision.

## Funding

This work was supported by the National Natural Science Foundation of China (No. 82360620, 82060008).

## Conflicts of Interest

The authors declare no conflicts of interest.

## Supporting information


**Figure S1:** qRT‐PCR analysis of *Pink1* and *Parkin* mRNA expression in mouse skin tissues. (A) qRT‐PCR analysis of *Pink1* mRNA expression in dorsal skin tissues. (B) qRT‐PCR analysis *Parkin* mRNA expression in dorsal skin tissues. The data are expressed as the mean ± SD. (*n* = 3) One‐way ANOVA with Student's *t*‐test was used to perform the comparison of means. *p < 0.05, **p < 0.01, compared to the Control group; ^#^ *p* < 0.05, ^##^ *p* < 0.01, compared with the AD group.

## Data Availability

The data that support the findings of this study are available from the corresponding author upon reasonable request.
