# Supplementary material for: Erianin Targets PINK1/Parkin‐Mediated Mitophagy and Apoptosis to Ameliorate Atopic Dermatitis
Source: Food Sci Nutr. 2026 Jul 24;14(7):e72158. doi: 10.1002/fsn3.72158 (PMC13400985; doi:10.1002/fsn3.72158)
Supplement: Supplementary file 1 — Figure S1: qRT‐PCR analysis of Pink1 and Parkin mRNA expression in mouse skin tissues. (A) qRT‐PCR analysis of Pink1 mRNA expression in dorsal skin tissues. (B) qRT‐PCR analysis Parkin mRNA expression in dorsal skin tissues. The data are expressed as the mean ± SD. (n = 3) One‐way ANOVA with Student's t‐test was used to perform the comparison of means. *p < 0.05, **p < 0.01, compared to the Control group; # p < 0.05, ## p < 0.01, compared with the AD group. [file FSN3-14-e72158-s001.docx]

**Supplementary materials**

**Supplementary methods:**

**2.11 Quantitative Real-Time RT-PCR**

Total RNA was extracted from mouse dorsal skin tissues using TRIzol reagent (Invitrogen, USA) following the manufacturer’s instructions. Complementary DNA (cDNA) synthesis was carried out via reverse transcription using the PrimeScript RT Reagent Kit (Takara, Japan). Real-time PCR was conducted with the LightCycler 480 System (Roche, Switzerland). Gene expression levels were normalized to mouse Gapdh, and relative gene expression was calculated using the 2^−ΔΔCt^ method. All primer sequences utilized in the analysis are detailed in Table 1.

**Supplementary Figure 1**


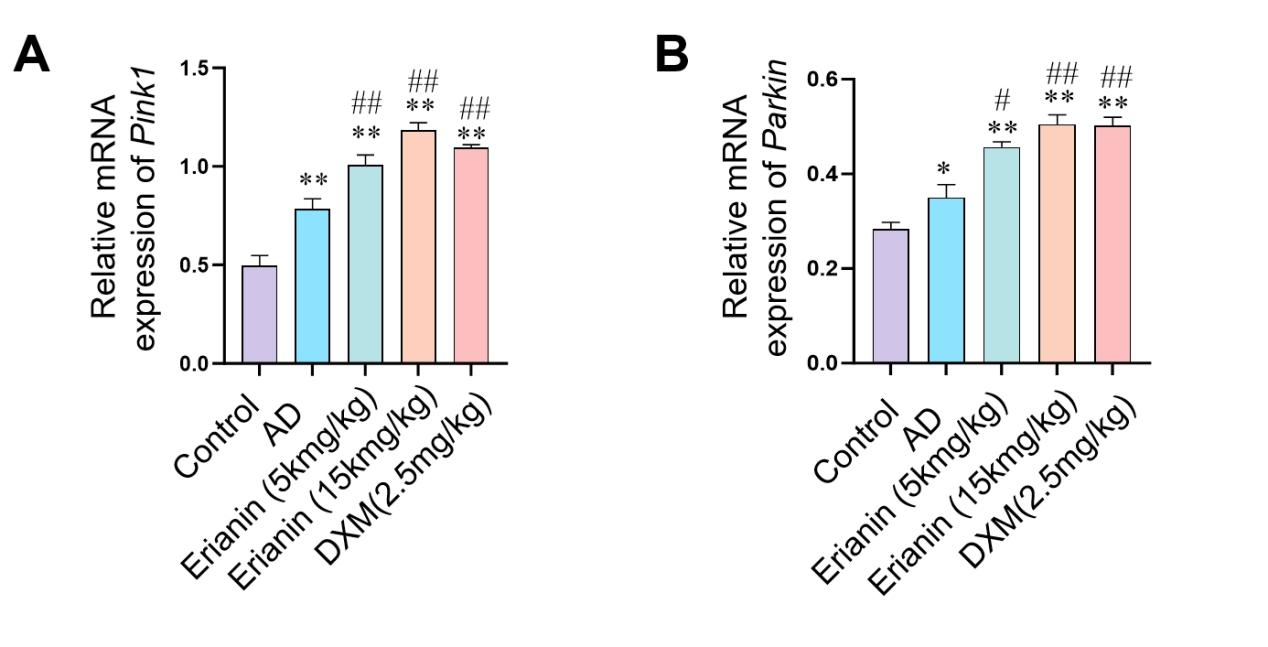


**Supplementary Figure legends**

Figure S1. qRT-PCR analysis of *Pink1* and *Parkin* mRNA expression in mouse skin tissues.

(A) qRT-PCR analysis of *Pink1* mRNA expression in dorsal skin tissues.(B) qRT-PCR analysis *Parkin* mRNA expression in dorsal skin tissues.The data are expressed as the mean ± SD.(n=3) One-way ANOVA with Student's t test was used to perform the comparison of means. **P* <0.05 ***P* <0.01, compared to the Control group; #*P* <0.05 ##*P* <0.01, compared with the AD group.
